# Early-Life Compartmentalization of Immune Cells in Human Fetal Tissues Revealed by High-Dimensional Mass Cytometry

**DOI:** 10.3389/fimmu.2019.01932

**Published:** 2019-08-14

**Authors:** Na Li, Vincent van Unen, Nannan Guo, Tamim Abdelaal, Antonios Somarakis, Jeroen Eggermont, Ahmed Mahfouz, Susana M. Chuva de Sousa Lopes, Boudewijn P. F. Lelieveldt, Frits Koning

**Affiliations:** ^1^Department of Immunohematology and Blood Transfusion, Leiden University Medical Center, Leiden, Netherlands; ^2^Leiden Computational Biology Center, Leiden University Medical Center, Leiden, Netherlands; ^3^Department of Pattern Recognition and Bioinformatics Group, Delft University of Technology, Delft, Netherlands; ^4^Department of Radiology, Leiden University Medical Center, Leiden, Netherlands; ^5^Department of Anatomy and Embryology, Leiden University Medical Center, Leiden, Netherlands

**Keywords:** imaging mass cytometry (IMC), immune composition, fetal intestine, fetal spleen, fetal liver, high-dimensional analysis, mass cytometry (CyTOF)

## Abstract

The human fetal immune system must protect the infant against the sudden exposure to a large variety of pathogens upon birth. While it is known that the fetal immune system develops in sequential waves, relatively little is known about the composition of the innate and adaptive immune system in the tissues. Here, we applied high-dimensional mass cytometry to profile the immune system in human fetal liver, spleen, and intestine. With Hierarchical Stochastic Neighbor Embedding (HSNE) we distinguished 177 distinct immune cell clusters, including both previously identified and novel cell clusters. PCA analysis indicated substantial differences between the compositions of the immune system in the different organs. Through dual t-SNE we identified tissue-specific cell clusters, which were found both in the innate and adaptive compartment. To determine the spatial location of tissue-specific subsets we developed a 31-antibody panel to reveal both the immune compartment and surrounding stromal elements through analysis of snap-frozen tissue samples with imaging mass cytometry. Imaging mass cytometry reconstructed the tissue architecture and allowed both the characterization and determination of the location of the various immune cell clusters within the tissue context. Moreover, it further underpinned the distinctness of the immune system in the tissues. Thus, our results provide evidence for early compartmentalization of the adaptive and innate immune compartment in fetal spleen, liver, and intestine. Together, our data provide a unique and comprehensive overview of the composition and organization of the human fetal immune system in several tissues.

## Introduction

The notion of phenotypical and functional differences between the fetal and adult immune system has been widely accepted. The human fetal immune system has to maintain tolerance toward the semi-allogeneic environment *in utero* while being prepared for the massive exposure to foreign antigens directly after birth (1, 2). The ontogeny of the immune system occurs in sequential waves during gestation. Fetal hematopoiesis is initiated in the yolk sac around day 16 of the development, then transits to the fetal liver at 6 weeks until 22 weeks gestational age, where the progenitors give rise to both lymphoid and myeloid cells (3). T cells have been identified as early as 10 weeks of gestation while Foxp3^+^CD4^+^ regulatory T (Treg) cells, whose generation is mainly driven by maternal alloantigens, have also been observed in different fetal tissues (4). Furthermore, it has been shown that human fetal dendritic cells in spleen, skin, thymus, and lung promote prenatal T-cell immune suppression (5). Interestingly, several studies have provided evidence for the existence of memory-like T (Tm) cells in fetal spleen (6), skin (7), intestine (8, 9), and cord blood (10), which produce pro-inflammatory cytokines such as IFN-γ and TNF-α, suggesting functional maturation of T cells *in utero*. In line with the discovery of pro-inflammatory T cells, mucosa-associated invariant T cells as well as natural killer (NK) cells and innate lymphoid cells (ILCs) have also been found to be present in the fetal intestine (11, 12). A potential link between the composition of the prenatal immune system and disease later in life has been proposed (9, 13). Thus, the fetal immune system has both pro-inflammatory and immune suppressive capacity.

Most investigations of the human fetal immune system are based on umbilical cord blood collected at birth. However, the representation of cord blood has recently been questioned as cord blood samples were heterogeneous without clearly shared patterns in cell and plasma protein composition (14). Although in recent years several reports (5, 8) have studied the fetal immune system in tissues, due to the scarcity of human fetal tissues and technique limitations, a system-wide and detailed characterization of the human fetal immune system is currently lacking. Mass cytometry (cytometry by time-of-flight; CyTOF) now offers the opportunity to analyze the heterogeneity of the human immune system in an unbiased and data-driven fashion by the simultaneous measurement of over 40 unique cellular markers at the single-cell level with unprecedented resolution (15). As traditional analysis approaches for flow cytometry are not compatible with high-dimensional mass cytometry datasets, dimensionality reduction-based approaches such as t-stochastic neighbor embedding (t-SNE) (16) have been widely used as they allow users to analyze all the markers concurrently in an unbiased manner. Moreover, hierarchical SNE (HSNE) has removed the scalability limitation of t-SNE, allowing the analysis of tens of millions of cells at single-cell resolution (17).

Here, we applied suspension mass cytometry to study the complexity and heterogeneity of the immune system in the human fetal intestine, liver, and spleen and confirmed the existence of previously identified subsets through the unsupervised HSNE analysis. In addition, we provide evidence for the existence of previously unrecognized distinct cell clusters. Besides the heterogeneity within each tissue, our data further reveals clear tissue-specific signatures in both the innate and adaptive immune compartment as early as week 16 of gestation. Finally, we employed imaging mass cytometry to reconstruct the tissue architecture and characterize and determine the location of the immune cell clusters within the tissue context, results that underpinned the compartmentalization of the immune system in the tissues. Together these data provide a comprehensive and valuable resource for understanding the fetal immune development and linking prenatal immunity with immunity after birth.

## Materials and Methods

### Human Fetal Tissues and Cell Isolation

Human fetal tissues from elective abortions were obtained from healthy pregnancy after informed consent. The gestational age ranged from 16 to 21 weeks. In total, 10 fetuses were included in the current study. Single-cell suspensions from different tissues were prepared as previously described (18). Briefly, the mesentery and meconium were removed from the fetal intestine. The intestines were then cut into small fragments and treated with 1 mM dithiothreitol (Fluka) in 15 mL of HBSS (Sigma-Aldrich) for 2 × 10 min (replacing buffer) at room temperature to dissolve the mucus and subsequently with 1 mM ethylenediaminetetraacetic acid (Merck) in 15 mL of Hank's balanced salt solution (ThermoFisher Scientific), under rotation for 2 × 1 h (replacing buffer) at 37°C to separate the epithelium from the lamina propria fraction. To obtain single-cell suspensions from the lamina propria, the intestines were rinsed with HBSS and incubated with 15 mL Iscove's Modified Dulbecco's Medium (IMDM; Lonza) supplemented with 10% fetal calf serum (FCS), 10 U/mL collagenase IV (Worthington), 200 μg/mL DNAse I grade II (Roche Diagnostics), at 37°C overnight, after which cell suspensions were filtered through a 70 μm nylon cell strainer. Finally, the immune cells were isolated with a Percoll (GE Healthcare) gradient. Fetal liver and spleen tissues were cut into small pieces then filtered through a 70 μm nylon cell strainer and the immune cells were isolated with Ficoll-Paque^TM^ density gradient (provided by the pharmacy of Leiden University Medical Center). All isolated cells were stored in liquid nitrogen. Study approval was granted by the Medical Ethics Commission of Leiden University Medical Center (protocol P08.087). All experiments were conducted in accordance with local ethical guidelines and the principles of the Declaration of Helsinki.

### Mass Antibodies and Antibody Conjugation

Antibodies used for suspension and imaging mass cytometry are listed in Tables S1, S3, separately. Conjugation of the purified antibodies lacking carrier protein with metal reporters was performed with the MaxPar X8 antibody labeling kit (Fluidigm Sciences) according to the manufacturer's instruction.

### Suspension Mass Cytometry Staining and Data Acquisition

Procedures for suspension mass cytometry antibody staining and data acquisition were carried out as previously described (18). Briefly, cells from different fetal tissues were incubated with 1 mL 500x diluted 500 μM Cell-ID intercalator-103Rh (Fluidigm Sciences) for 15 min at room temperature to identify dead cells. Cells were then stained with metal-conjugated antibodies for 45 min at room temperature. After staining, cells were labeled with 1 mL 1,000x diluted 125 μM Cell-ID intercalator-Ir (Fluidigm Sciences) to stain all cells in MaxPar Fix and Perm Buffer (Fluidigm Sciences) overnight at 4°C. Finally, cells were acquired by CyTOF 2^TM^ mass cytometer (Fluidigm Sciences). Data were normalized by using EQ Four Element Calibration Beads (Fluidigm Sciences) with the reference EQ passport P13H2302 during the course of each experiment.

### Imaging Mass Cytometry Staining and Data Acquisition

Snap-frozen human fetal splenic, intestinal and liver biopsies were sectioned at a thickness of 5 μm. All sections were fixed by incubating with 1% paraformaldehyde for 5 min at room temperature followed by 100% methanol for 5 min at −20 °C. After fixation, tissue sections were washed in Dulbecco's phosphate-buffered saline (ThermoFisher Scientific) containing 0.5% bovine serum albumin and 0.05% Tween, rehydrated in additive-free Dulbecco's phosphate-buffered saline. After washing again, tissue sections were blocked with Superblock Solution (ThermoFisher Scientific) for 30 min in a humid chamber. Tissue sections were then stained with a metal-conjugated antibody master mix overnight at 4°C, washed and incubated with 125 nM Cell-ID Intercalator-Ir for 30 min at room temperature. After a further wash, tissue sections were dipped in Milli-Q water (Merck Millipore) for 1 min and dried for 20 min at room temperature. The acquisition was performed using a Hyperion imaging mass cytometer (Fluidigm Sciences) at a resolution of 1 μm, with settings aligned to company guidelines. The ablation frequency was 200 Hz, and the energy was 6 dB. Regions of interest were acquired at a size of 1 × 1 mm^2^. All data were stored as MCD files and txt files.

### Data Analysis

**Figure 1:**
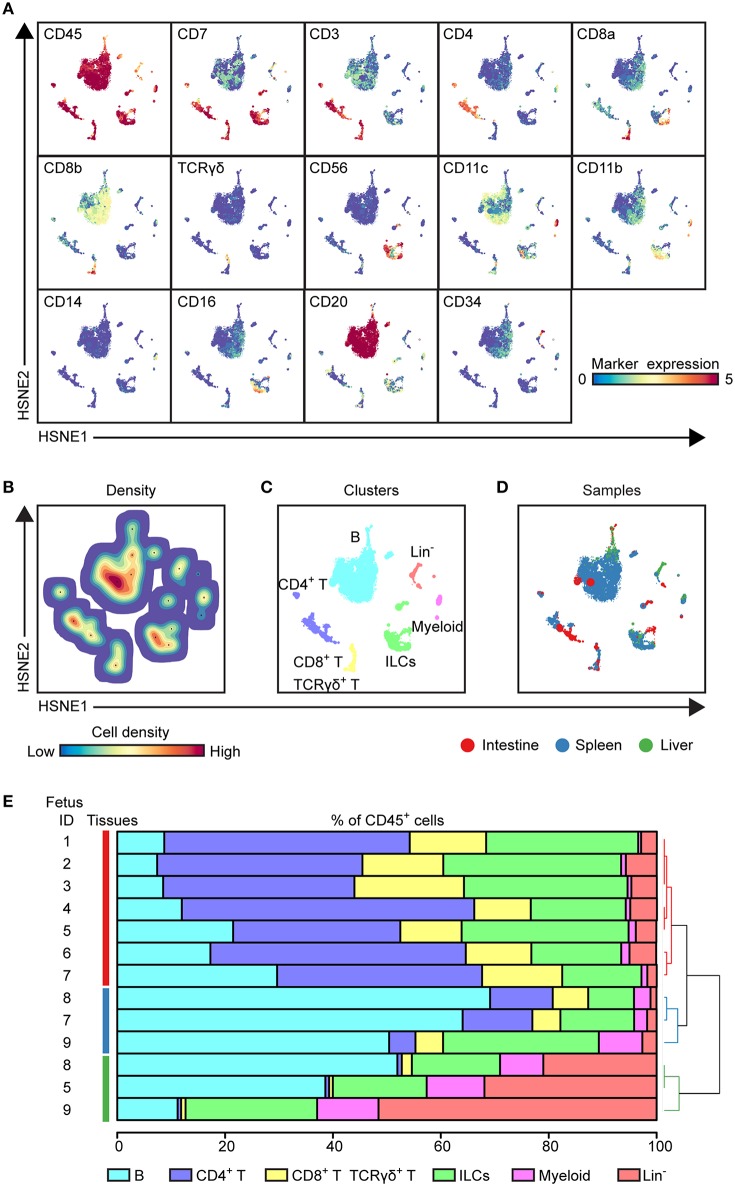
HSNE analysis distinguishes major immune lineages across human fetal tissues. **(A)** HSNE embedding of 1.6 million immune cells derived from fetal intestines (*N* = 7, 0.3 × 10^6^ cells), fetal spleens (*N* = 3, 1.1 × 10^6^ cells), and fetal livers (*N* = 3, 0.2 × 10^6^ cells) at the overview level. Each dot represents a HSNE landmark and the size of the landmark is proportional to the number of cells that each landmark represents. Colors of the landmarks represent ArcSinh5-transformed expression values of the indicated markers. **(B)** A density map showing the local probability density of the embedded cells where black dots display the centroids of identified clusters using GMS clustering. **(C)** A HSNE plot showing main immune lineage cluster partitions in different colors. **(D)** HSNE embedding as shown in **(A)**. Colors represent different tissues. **(E)** The composition of major immune lineage clusters for CD45^+^ cells in the individual fetal tissues is represented in horizontal bars where the colored segment lengths represent the proportion of cells as a percentage of CD45^+^ cells in the sample. The dendrogram shows the hierarchical clustering of samples. Colors represent the different tissues as shown in **(D)**. Numbers indicate fetus ID.

Data for single, live CD45^+/dim^ cells gated from each sample individually using Cytobank as shown in Figure S1, were sample tagged and hyperbolic arcsinh transformed with a cofactor of 5 within Cytosplore^+HSNE^ (17). The major immune lineages in Figure 1 were then identified by performing hierarchical stochastic neighbor embedding (HSNE) in Cytosplore^+HSNE^ software (17). HSNE was carried out with default parameters (perplexity: 30; iterations: 1,000). For the cluster identification, each cluster contains at least 100 cells. All HSNE plots were generated in Cytosplore (19). Cellular signatures of immune cells for each sample individually were generated in Cytosplore. Due to homogeneity and abundance of B cells in the fetal spleen, B cells from each spleen were downsampled to 50,000 cells to deduce the cellular signatures. The similarity between two paired t-SNE maps was quantified by Jensen-Shannon (JS) divergence by measuring the similarity between corresponding probability density distribution after converting t-SNE maps to two-dimensional probability density functions in Matlab R2015b. Hierarchical clustering of the phenotype heatmap was created with Euclidean correction and average linkage clustering while the cell frequency heatmap with Spearman correction and average linkage clustering in Matlab R2015b. Principal component analysis (PCA) was computed with the cluster frequencies of CD45^+/dim^ cells in the individual samples using “prcomp” function, and the result was visualized using “ggbiplot” function in R software. The cluster t-SNE map in **Figure 5B** was performed as our previous study (20). Briefly, the data matrix with cluster frequencies of CD45^+/dim^ cells in the individual samples as input variance was normalized and computed to select the top ten highest variance principal components as input to the t-SNE analysis. Hence, the cluster with similar profiles clustered together in the t-SNE map. Images in **Figure 6** and Figure S4 were generated using MCD Viewer software v1.0.560 (Fluidigm Sciences).

## Results

### Identification of Major Immune Lineages Across Human Fetal Tissues

To explore the immune system in the human fetus, we employed a previously described CyTOF panel (Table S1) consisting of 35-metal isotope-tagged monoclonal antibodies (18) designed to identify the major immune lineages (B cells, CD4^+^ T, CD8^+^ T, γδ T cells, ILCs, and myeloid cells) and determine the heterogeneity within these lineages. For this purpose, the panel consisted of lineage markers, markers specific for cell differentiation, activation, trafficking, and function. With this panel, single-cell suspensions from fetal intestines (*N* = 7), fetal spleens (*N* = 3), and fetal livers (*N* = 3) Table S2) were analyzed. Single, live CD45^+^ cells were distinguished by event length, DNA stains, and CD45 antibody stains (Figure S1A). In the liver, but not in the spleen and intestine, three distinct subpopulations were observed based on different CD45 and DNA stainings (Figure S1B). Here, CD45^hi^DNA^low^ cells represent lymphoid cells, while CD45^hi^DNA^hi^ and CD45^low^DNA^hi^ cells correspond to myeloid and CD34^+^ precursor cells, respectively (Figure S1B). All antibodies displayed a clear separation between antibody-negative and -positive cells as described previously (18).

To determine the major immune lineages, we pooled the data (1.6 × 10^6^ CD45^+^ cells) derived from seven fetal intestines (39,357 ± 17,836 cells), three fetal spleens (365,653 ± 148.098 cells) and three fetal livers (69,928 ± 18,146 cells) (Figure S1C) and performed a 3-level HSNE analysis in Cytosplore^+HSNE^ (17) from a global overview down to the single cell level. Here, HSNE landmarks depicted the global composition of the immune system (Figure 1). Based on the marker expression profiles (Figure 1A) and density features of the embedded cells (Figure 1B), we identified 6 phenotypically distinct major lineage clusters at the overview level, namely CD20^+^ B cells, CD3^+^CD4^+^ T cells, CD3^+^CD8^+^ T cells and CD3^+^TCRγδ^+^ T cells, CD3^−^CD20^−^CD11c^−^CD7^+^ ILCs, CD11c^+^ myeloid cells and a CD3^−^CD20^−^CD11c^−^CD7^+/−^ lineage-negative (Lin^−^) cell cluster (Figure 1C). In addition, the global differences in immune composition across tissues were revealed by visualizing the tissue-origin of the cells (Figure 1D). Next, we quantified the relative frequencies of major immune lineage clusters in each fetal tissue sample, showing that B cells were more abundant in the fetal spleen and fetal liver while Lin^−^ cells were most profound in the fetal liver. CD4^+^ T, CD8^+^ T, and γδ T cells together comprised more than 50% of immune cells in the fetal intestine but were typically lower in the fetal liver. There was no significant difference in the distribution of ILCs across fetal tissues. The frequencies of myeloid cells were low in all fetal tissues, however, these cells were relatively more abundant in fetal liver (Figure 1E). Importantly, unbiased hierarchical clustering of cell frequencies grouped the tissue samples in a tissue-specific manner (Figure 1E), indicating that despite differences between the samples the composition of the major immune lineage of each fetal tissue was relatively constant and tissue-specific.

Together, these global analyses revealed that the major immune lineages could be readily identified in each fetal tissue, and that the composition of these lineages differs between the examined human fetal tissues.

### Visualization of Cellular Signatures Across Human Fetal Tissues

**Figure 2:**
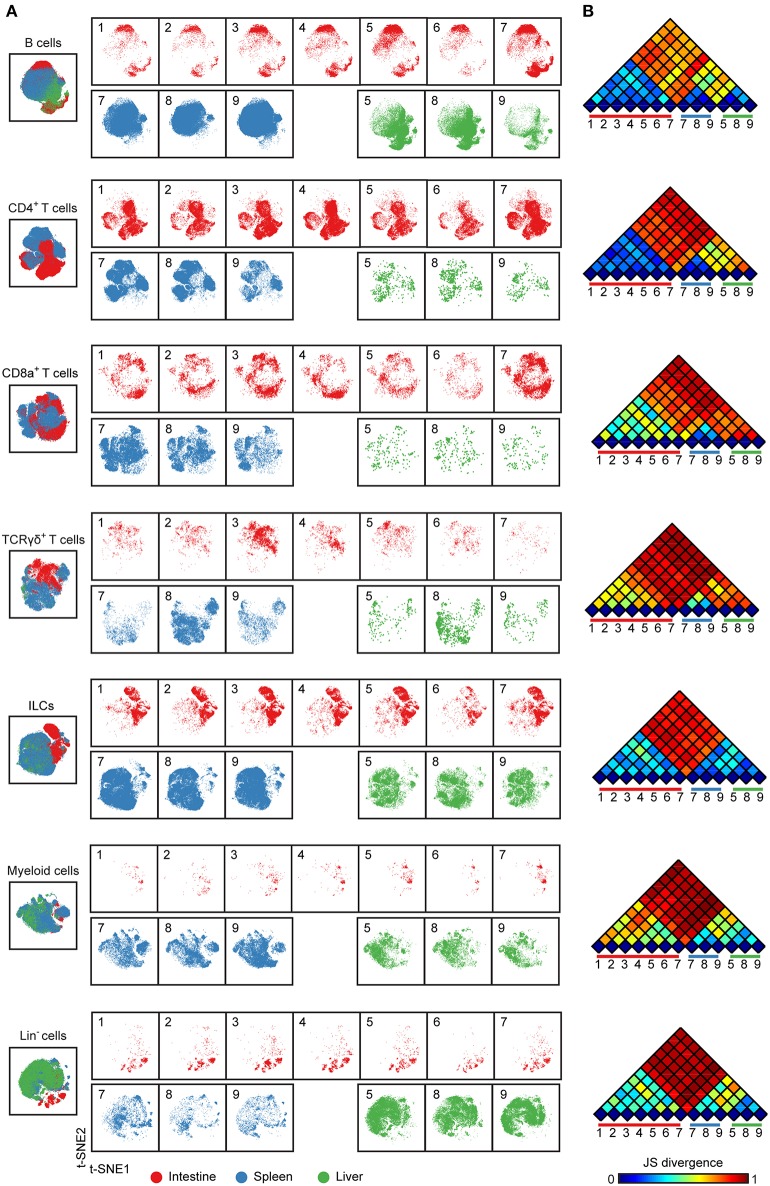
Cellular signatures reveal the immune cell compartmentalization across human fetal tissues. **(A)** A collective t-SNE was performed on each major immune lineage individually and stratified for samples. The plots are showing in total 1.5 × 10^5^ B cells, 2.3 × 10^5^ CD4^+^ T cells, 6.9 × 10^4^ CD8^+^ T cells, 3.6 × 10^4^ γδ T cells, 2.6 × 10^5^ ILCs, 6.4 × 10^4^ myeloid cells and 8.7 × 10^4^ Lin^−^ cells. Colors represent different tissues. Numbers indicate fetus ID. **(B)** The similarity of a pair of t-SNE plots is shown by Jensen-Shannon (JS) divergence within the major immune lineage. A higher JS divergence value indicates higher dissimilarity between pairwise t-SNE plots.

To compare immune cells between all samples and between the tissues, we next selected the major immune lineage clusters individually and embedded the clusters at the single-cell level to deduce the cellular signatures (Figure 2) (20). The results demonstrate that the intestine, spleen, and liver displayed a distinct cellular signature for most of the seven major immune lineages. We observed especially highly distinct differences between the intestinal samples compared to the spleen and liver samples (Figure 2A). Also, the results underscore that samples from the same tissue type have a highly similar cellular signature (Figure 2A). We next applied the Jensen-Shannon (JS) divergence method to quantify the similarities and differences between pairs of t-SNE maps (Figure 2B). For all major immune lineages, the JS divergence was highest between the intestinal samples and the spleen and liver samples, while considerable differences in JS divergence were also found between the spleen and the liver samples, except for the ILC compartment. In general, the composition of the B cells, CD4^+^ T cells and ILCs was more similar within a tissue group while more variation was present among CD8^+^ T cells, γδ T cells, and myeloid cells (Figure 2B). Together, the visualization of cellular signatures provided further evidence for the distinct composition of immune system in the fetal tissues.

### High-Dimensional Analysis Reveals Unprecedented Immune Heterogeneity in Human Fetal Tissues

**Figure 3:**
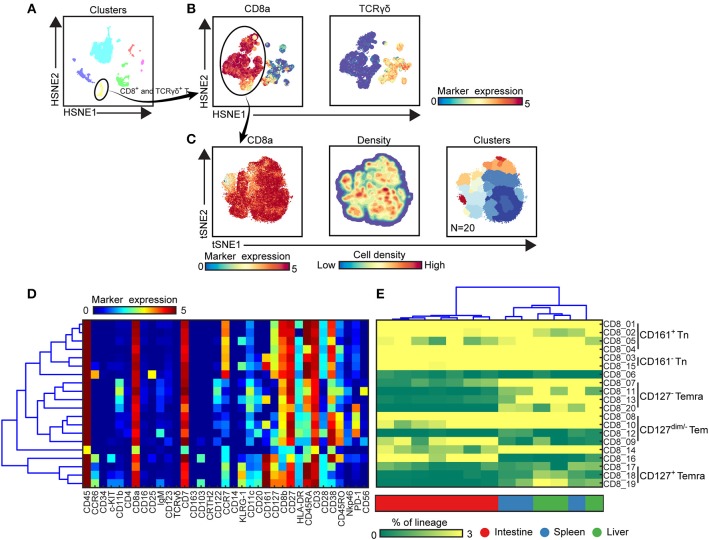
Identification of phenotypically distinct clusters in the CD8^+^ T cell compartment across human fetal tissues. **(A)** Global level of HSNE embedding of 1.6 million immune cells as shown in Figure 1C showing major immune lineage cluster partitions in different colors. **(B)** Second level of HSNE embedding of CD8^+^ T and γδ T cells (1.1 × 10^5^ cells) derived from fetal intestines (*N* = 7), fetal spleens (*N* = 3), and fetal livers (*N* = 3). Colors represent the ArcSinh5-transformed expression values of CD8a and TCR γδ. **(C)** A t-SNE embedding of collective CD8^+^ T cells (6.9 × 10^4^ cells) derived from fetal intestines (*N* = 7), fetal spleens (*N* = 3), and fetal livers (*N* = 3). Colors represent the ArcSinh5-transformed expression values of CD8a (left panel). A density map showing the local probability density of the embedded cells (middle panel). Colors represent cluster partitions (right panel). **(D)** Heatmap (blue-to-red scale) showing the median marker expression values of the clusters identified in **(C)** and hierarchical clustering thereof. **(E)** Heatmap (green-to-yellow scale) showing the corresponding cell frequencies of identified clusters of total CD8^+^ T cells in each sample. The dendrogram shows the hierarchical clustering of samples. Colors represent different fetal tissues. Tn, naive T cells; Tem, effector memory T cells; Temra, terminally differentiated T cells.

To extend the analysis we next selected every main immune lineage individually and embedded them at the second level (B cells) or single-cell data level to identify the phenotypically distinct clusters, here illustrated for the CD8^+^ T cell compartment (Figure 3). First, the cluster including CD8^+^ T and γδ T cells (105,211 cells) were selected at the overview level and embedded in the second level of the HSNE analysis (Figures 3A,B), followed by selection of the CD8^+^ T cells (69,269 cells) to zoom-in further (Figure 3B), revealing more single-cell details (Figure 3C). Based on the density features of the t-SNE-embedded cells, we identified 20 distinct CD8^+^ T cell clusters (Figure 3C), each defined by a unique maker expression profile (Figure 3D). After hierarchical clustering of the heatmap, these clusters grouped into five main metaclusters (Figures 3D,E). CD45RA^+^CCR7^+^ naive T (Tn) cells were classified into two groups based on CD161 expression while CD45RA^−^CCR7^+^ central memory T (Tcm) and CD45RA^+^CCR7^−^ terminally differentiated T (Temra) cells were detected in the CD127^dim/−^ compartment. In addition, a group of CD127^+^CD161^high^CCR6^+^KLRG-1^+^ Temra cells clustered separately from the other groups. We next quantified the cell frequencies of each cluster per sample and performed the hierarchical clustering of different samples based on the cell frequencies. In line with previous studies (21), several clusters including Tn cells (CD8_01-05, 15) and Tem cells (CD8_08, CD8_10) were present in all tissues (Figure 3E). CD127^−^CD161^+/−^ Temra (CD8_07, CD8_11, CD8_13, and CD8_20) mainly existed in the spleen and liver whereas PD-1^−^ Tem (CD8_09) were mainly abundant in the intestine. Hierarchical clustering revealed that all the intestinal samples clustered together whereas the spleen and liver samples clustered intermixed with each other.

**Figure 4:**
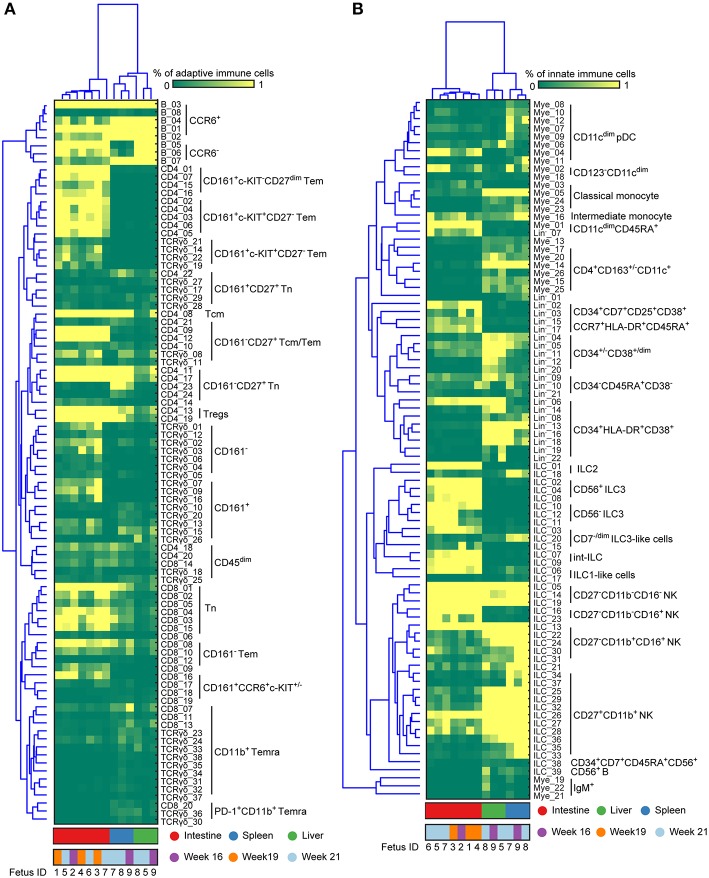
Immune system landscapes visualize cell cluster composition across human fetal tissues. **(A,B)** Heatmap showing the cell frequencies of 90 adaptive and 87 innate immune clusters identified in Figures S2, S3 combined with hierarchical clustering of samples (Top) and phenotype of clusters (Left). Colors represent different fetal tissues as indicated. Tem, effector memory T cells; Tcm, central memory T cells; Temra, terminally differentiated T cells; pDC, plasmacytoid dendritic cells.

By applying this approach to all 7 major immune lineage clusters, we identified 177 phenotypically distinct clusters (8 B cell clusters, 24 CD4^+^ T cell clusters, 20 CD8^+^ T cell cluster, 38 γδ T cell cluster (Figures S2A–C and Figure 4A), 39 ILC clusters, 26 myeloid cell clusters and 22 Lin^−^ clusters (Figures S3A,B and Figure 4B). Consistent with previous reports (2), IgM^+^HLA-DR^+^ B cells were identified in all three tissues while the plasmacytoid dendritic cells (pDC)-like cells and monocytes were mainly identified in the intestine and non-intestine, respectively. In addition to Tn cells, we also identified several clusters of Tm cells, which were more prominent in the CD4^+^ T cell compartment in the fetal intestine, as reported (9). Strikingly, some of these Tm cells expressed a higher level of CD127, CD161, CCR6 and c-KIT, the latter a marker typically associated with progenitor cell types. Similarly, we identified a rare c-KIT^+^ (CD117^+^) CD8^+^ T cell population (CD8_16, <1% of CD45^+^ cells) that is almost exclusively present in the intestine. As reported (22), helper-ILCs were more dominant in the fetal intestine than the fetal spleen and liver, where the majority of ILCs were different types of NK cells such as CD27^−^CD11b^+^, CD27^+^CD11b^+^ NK clusters. Moreover, CD34^+^CD38^+^, CD34^+^CD38^dim^, CD34^+^CD38^−^ precursor cell clusters and several previously unknown precursor-like cell clusters were mainly identified in the fetal liver (e.g., Lin^−^_02, Lin^−^_03, Lin^−^_15, Lin^−^_17) (Figure 4), an important organ for fetal hematopoiesis. Unbiased hierarchical clustering of cell frequencies for each sample revealed a clear distinction between the fetal intestine and the other two tissues in both the adaptive and innate immune compartment (Figure 4). The separation between fetal spleen and liver was readily observed in the innate immune compartment, due to the differential abundance of CD34^+^ cells and certain types of ILCs, such as ILC2 and CD7^dim^ ILC3-like cells (Figure 4B), but not in the adaptive immune compartment.

Altogether, our data reveal that there is far greater heterogeneity of the immune system in human fetal tissues than previously appreciated and provide evidence for the existence of previously unknown immune cell types.

### An Integrated System-Wide Analysis of the Immune System Reveals Early Immune Compartmentation Across Fetal Tissues

**Figure 5:**
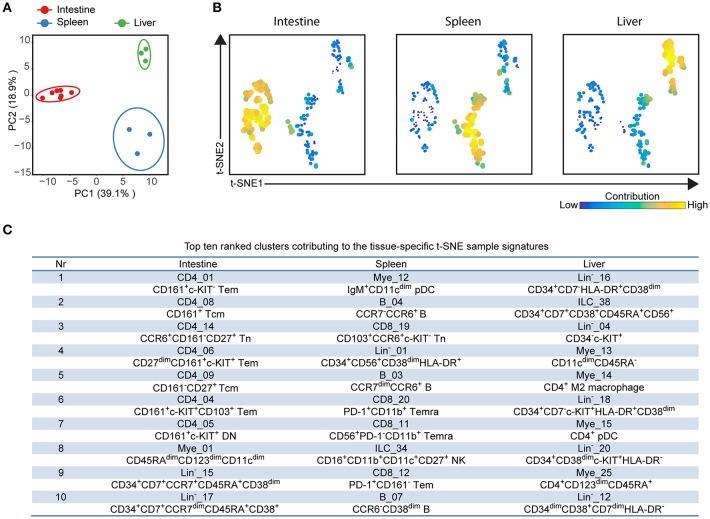
Integrated analysis of immune cluster composition reveals highly discriminatory tissue-specific signatures. **(A)** Principal component analysis (PCA) of 13 fetal samples. PCA was computed on the cell frequencies of 177 immune clusters per sample (as the percentage of CD45^+^ cells) and top 2 principal components are shown describing 58% of total variance. One dot represents a single sample. Colors represent different fetal tissues. **(B)** t-SNE embedding of 177 immune clusters derived from 13 samples showing the tissue-specific signatures. Every dot represents a single immune cluster. The size of the dot is proportional to the value of cell frequency, which is more similar across tissues when the clusters are together closer. **(C)** Table depicting top ten ranked clusters contributing to tissue-specific t-SNE sample signatures with biological annotation. Tem, effector memory T cells; Tcm, central memory T cells; Temra, terminally differentiated T cells; pDC, plasmacytoid dendritic cells; NK, natural killer cell.

In order to investigate the entire immune profiles across fetal tissues, a PCA was performed on the samples based on the cluster frequency values of CD45^+/dim^ cells. The samples were clearly separated from each other based on the tissue origin, especially the intestines and the other two organs by principal component 1 (explaining 39.1% variance) (Figure 5A). In consistency with the cellular signatures (Figure 2), the variance of immune composition within spleens was higher than that within intestines and livers (Figure 5A).

To reveal which cell clusters were most strongly associated with the tissue-specificity, we performed a t-SNE analysis on 177 clusters based on the frequency values, visualizing networks of cell clusters, which determined the tissue-associated patterns (Figure 5B). Moreover, the dual-tSNE identified the top ten ranked clusters that contributed to the sample clustering patterns (Figure 5C). This analysis yielded three distinct networks of cell clusters corresponding to the three tissue types (Figure 5B). Several distinct hematopoietic progenitor clusters (Lin^−^_16, ILC_38, Lin^−^_04, Lin^−^_18, Lin^−^_20, and Lin^−^_12) and four myeloid populations (Mye_13, Mye_14, Mye_15, and Mye_25) were specifically found in the fetal liver. For the fetal intestines, the main types of contributing cells were lymphoid precursors (Lin^−^_15 and Lin^−^_17) and CD4^+^ Tm clusters (CD4_01, CD4_08, CD4_09, CD4_06, CD4_04, CD4_05), including three previously unidentified c-KIT^+^ clusters, while for the spleen a diverse pool of CD8^+^ T cells, B cells and myeloid cells contributed to the tissue-specific signature (Figure 5C). Together, the integrated system-wide analysis of the immune system reveals a unique immune cell landscape and early-life immune compartmentation across human fetal tissues, suggesting different immune responses *in situ* during human fetal development.

### Imaging Mass Cytometry Reveals the Tissue-Specific Signatures *in situ*

To extend the understanding of the fetal immune system, we applied an imaging mass cytometry panel comprising 31 antibodies to determine the spatial distribution of the immune and stromal cells in the tissues *in situ* from one individual. This panel contained markers for visualizing the tissue architecture such as E-cadherin (epithelium), D2-40 (lymphatic endothelium), vimentin (intermediate filament), smooth muscle actin (SMA) and collagen I (extracelluar matrix), markers to identify various immune cell subsets such as T cells (CD3, CD8, CD4, CD45RA, CD45RO), NK cells (CD7 and CD56), myeloid cells (CD163) and antigen presenting cells/activated cells (HLA-DR), as well as markers expressed by both stromal and immune cells such as CD31. In addition, the proliferation marker Ki-67 was included (Table S3). With this panel, tissue sections derived from the fetal intestine, spleen and liver were analyzed. All antibodies showed clear positive signals as illustrated in one of the regions of interest (ROIs) derived from the fetal intestine (Figure S4).

**Figure 6:**
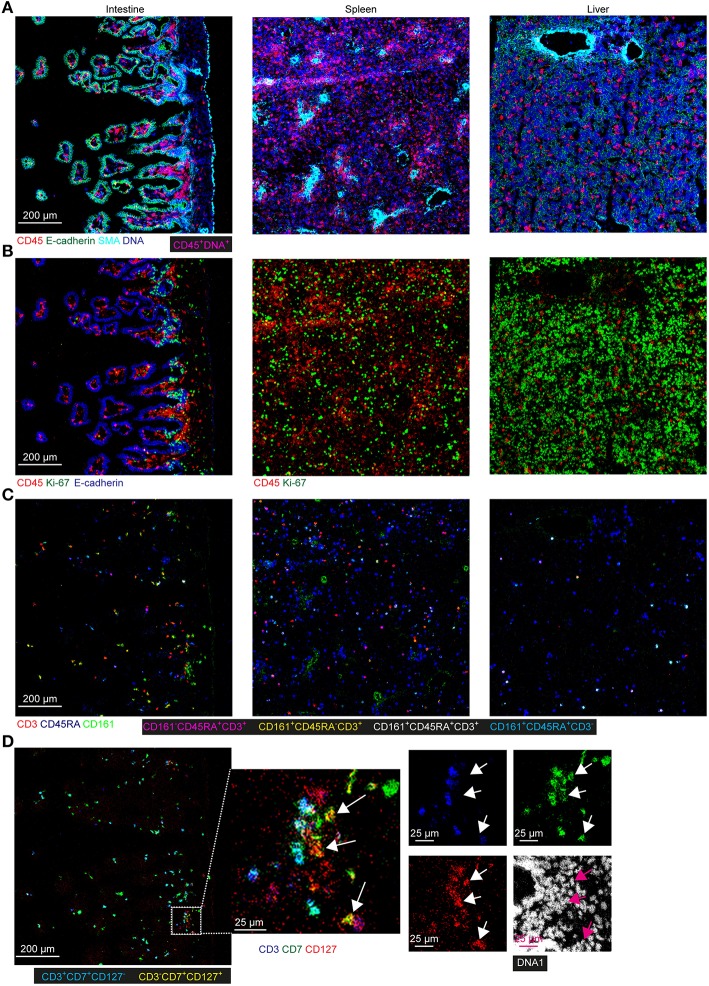
Spatial localization of immune and stromal cell subsets across tissues. **(A–D)** Representative mass cytometry images of one region of interest (ROI) of the fetal intestine, spleen, and liver derived from one individual, displaying the overlay of the indicated makers. Scale bar: 200 μm. Colors and scale bars are identical in **(A–C)**. The numbers of ROIs for intestine, spleen, and liver are 6, 2 and 4, respectively. **(A)** CD45 in red, E-cadherin in green, SMA in cyan and DNA in blue. **(B)** CD45 in red, Ki-67 in green and E-cadherin in blue. **(C)** CD3 in red, CD45RA in blue and CD161 in green. **(D)** CD3 in blue, CD7 in green, CD127 in red and DNA in white. White and purple arrows indicate the CD3^−^CD7^+^CD127^+^ ILCs.

To obtain further information on the phenotype and localization of immune cell subsets within the tissue context, we visualized combinations of specific markers (Figure 6). Staining with E-cadherin, SMA and DNA were used to show the tissue structure, reflecting the overall morphological differences between the tissues and the distribution of the immune cells was revealed by CD45 staining. Most CD45^+^ immune cells were in the E-cadherin^−^ lamina propria in the intestine whereas these cells were scattered in the fetal spleen and liver (Figure 6A). While the majority of CD45^+^ immune cells lacked expression of Ki-67 in all three tissues, many non-immune cells in the fetal liver and to a lesser extent in the fetal spleen were stained by the Ki-67-specific antibody, indicative of cell division and likely linked to the development of these organs at this stage (Figure 6B). In agreement with earlier work, Ki-67^+^ cells in the intestines resided in the crypt (Figure 6B), where stem cells and progenitor cells are enriched (23). Consistent with findings using suspension mass cytometry, both CD45RA^+^ Tn and CD45RA^−^ Tm cells were identified in all three tissues. Compared with the spleen and liver, the CD45RA^−^ Tm cells were most abundant in the intestine, especially CD161^+^CD45RA^−^ T cells (Figure 6C). CD161^−^CD45RA^−^ T cells and CD161^+^CD45RA^+^ T cells were more predominate in the spleen and liver as compared with the other two tissues, respectively (Figure 6C). Finally, simultaneous visualization of CD3, CD7, and CD127 reveals that CD3^+^ T cells and CD3^−^CD7^+^CD127^+^ ILCs (Figure 6D, white and purple arrows) were located in close proximity of each other in the lamina propria of the intestine.

Together, these data confirmed the existence of tissue-specific subsets *in situ* and underpinned the distinctness of the immune system in the tissues at the second trimester during pregnancy.

## Discussion

While recent studies have investigated the heterogeneity and functionality of the developing immune system in prenatal life (5, 9, 10), knowledge of the immune system development remains sparse due to the relative scarcity of fetal tissues. In the current study, we applied high-dimensional mass cytometry to dissect both the innate and adaptive immune compartment in the human fetal intestine, spleen, and liver in an unbiased and data-driven manner. For data analysis, we used HSNE (17), an in-house developed software, which allows the identification of phenotypically distinct clusters in datasets consisting of millions of cells. In line with previous work (5, 6, 9, 10, 22), we were able to readily define the major cell lineages and substantial heterogeneity therein, and reveal the identity of previously unrecognized cell clusters. Moreover, our results provide evidence for tissue-specific compartmentalization of both the innate and adaptive immune compartment early in the second trimester of pregnancy. Altogether, our results confirm and extend previous studies that have analyzed the immune subsets in mucosal and lymphoid tissues from human fetus with immunohistochemical analysis (8, 24) or traditional multi-color flow cytometry (5, 25). Our comprehensive analysis provides a valuable resource that may aid future studies into the development of the human immune system during gestation.

Consistent with previous reports, our analysis confirmed the distinctness of the human fetal liver, with enrichment of myeloid and CD34^+^ precursor cell clusters. Six out of the top ten ranked clusters contributing to liver-specific signatures were CD34^+^ precursor cells. Also, we observed extensive diversity within the CD34^+^ cell population due to differential expression of several markers including c-KIT and HLA-DR, possibly reflecting differentiation pathways and linked to the crucial role of the fetal liver in haematopoiesis in prenatal life.

In addition, several other features distinguished the immune systems found in the tissues examined. Here, CD27^−^CD11b^−^ NK cells exhibiting dominant inhibitory signals (26) were found primarily in the intestine while CD27^−^CD11b^+^ NK and CD27^+^CD11b^+^ NK cells with dominant activating signals (26) predominate in the spleen and liver and contribute to the tissue partitions and demarcate different functions in the tissues examined. Also, helper-ILCs were mainly present in the fetal intestine, as reported (22). Moreover, we have previously reported that memory-like T cells are located in the fetal intestine (9) but to a much lower degree in fetal spleen and liver indicative of *in situ* immune priming and maturation of T cells in the developing intestine. Intriguingly, previously unidentified CCR6^+^c-KIT^+^CD45RO^+^ subpopulations of T cells were identified in all lineages in the fetal intestine, but not in other organs. Expression of c-KIT, a receptor for stem cell factor, has been shown to demarcate a subset of human CD8^+^ Tm cells with self-renewal properties (27). Moreover, flow-sorted c-KIT^+^ Tm cells cocultured with OP9 stromal cells in the presence of stem cell factor and IL-7 proliferated *in vitro* (data not shown). These observations suggest a potential role for CCR6^+^c-KIT^+^ Tm cells in the conservation of mucosal T cell memory.

CD161 has previously been shown to define a transcriptional and functional phenotype across human T cell lineages with an innate-like ability to respond to cytokines (28) while another study has described that CD161 mediates prenatal immune suppression (29). In our study, CD161-expressing T cells are more pronounced in the human fetal intestine as compared to fetal spleen and liver, consistent with a unique function of CD161^+^ cells within the fetal intestine. HLA-DR^+^ myeloid cells and B cells were found in all three organs, however, all B cells lacked expression of CD27, a classical memory B cell marker.

We have used imaging mass cytometry to gain insight into the localization of specific immune subsets within the tissue context. The results reiterate the highly distinct organization of the immune compartment in the three tissues investigated and allow the simultaneous analysis of cellular activity, i.e., cell proliferation. We demonstrate that this can be used to confirm results obtained by single-cell suspension mass cytometry while simultaneously obtaining information on the co-localization of specific immune subsets. For example, we obtained evidence that in the intestinal lamina propria CD3^+^ T cells are found in close proximity of CD3^−^CD7^+^CD127^+^ ILC, possibly pointing toward “crosstalk” between these cell subsets. This will be the topic of future studies.

While this study is one of the first to study the immune landscape in human fetal tissues with high-dimensional analysis at the single-cell level, our study has limitations. First, we did not observe much heterogeneity in the B population as our antibody panel lacked several markers required for this. It would thus be important to include other antibodies such as IgD, CD10 and CD5 to dissect the B compartment further. Second, we had only access to fetal material from the second trimester (16–21 gestational weeks) and obtained only a limited number of fetal tissues. In future studies, it would thus be valuable also to include samples from the first and third trimester and increase the number of fetal tissues. Third, while we identified 177 known and unknown cell clusters, the functional properties of these subsets need to be clarified in future studies. Also, as we did notice some minor differences in the composition of the myeloid compartment when comparing tissue samples of different gestational age, future studies should address this in more detail. Finally, due to the cell isolation procedure the dataset does not provide information on granulocytes and red blood cells. It will be valuable to investigate this in future studies. It should be noted that while the analysis provides comprehensive insight into the organization of the tissue sample investigated, a single specimen biopsy cannot reflect the structural complexity of these organs, this will be the subject of future studies.

Nevertheless, our mass cytometric analysis provides the first global, comprehensive, and detailed description of the immune landscape in the developing fetus in several tissues, reveals tissue-specific signatures, and demonstrates a clear immune compartmentalization in the tissues in prenatal life. Recent studies have indicated that events shaping the immune system *in utero* and in early life can have a significant impact on the development of diseases in adult life (30–32). Detailed understanding of the early development of the immune system is crucial for the development of strategies to prevent such diseases and our study may help to achieve that goal.

## Data Availability

Mass cytometry data are available via Flow Repository (https://flowrepository.org/id/FR-FCM-Z26T).

## Ethics Statement

Human fetal tissues from elective abortions were obtained after informed consent. Study approval was granted by the Medical Ethics Commission of Leiden University Medical Center (protocol P08.087). All experiments were conducted in accordance with local ethical guidelines and the principles of the Declaration of Helsinki.

## Author Contributions

NL and FK conceived the study and wrote the manuscript. NL performed most experiments with the help of NG. NL performed most of the analyses with the help of VvU, TA, AS, JE, AM, and BL. SC provided human fetal tissues. All authors discussed the results and commented on the manuscript.

### Conflict of Interest Statement

The authors declare that the research was conducted in the absence of any commercial or financial relationships that could be construed as a potential conflict of interest.
