# Supplementary material for: Early-Life Compartmentalization of Immune Cells in Human Fetal Tissues Revealed by High-Dimensional Mass Cytometry
Source: Front Immunol. 2019 Aug 14;10:1932. doi: 10.3389/fimmu.2019.01932 (PMC6703141; doi:10.3389/fimmu.2019.01932)
Supplement: Supplementary file 1 [file Data_Sheet_1.pdf]

Supplementary figures and figure legends

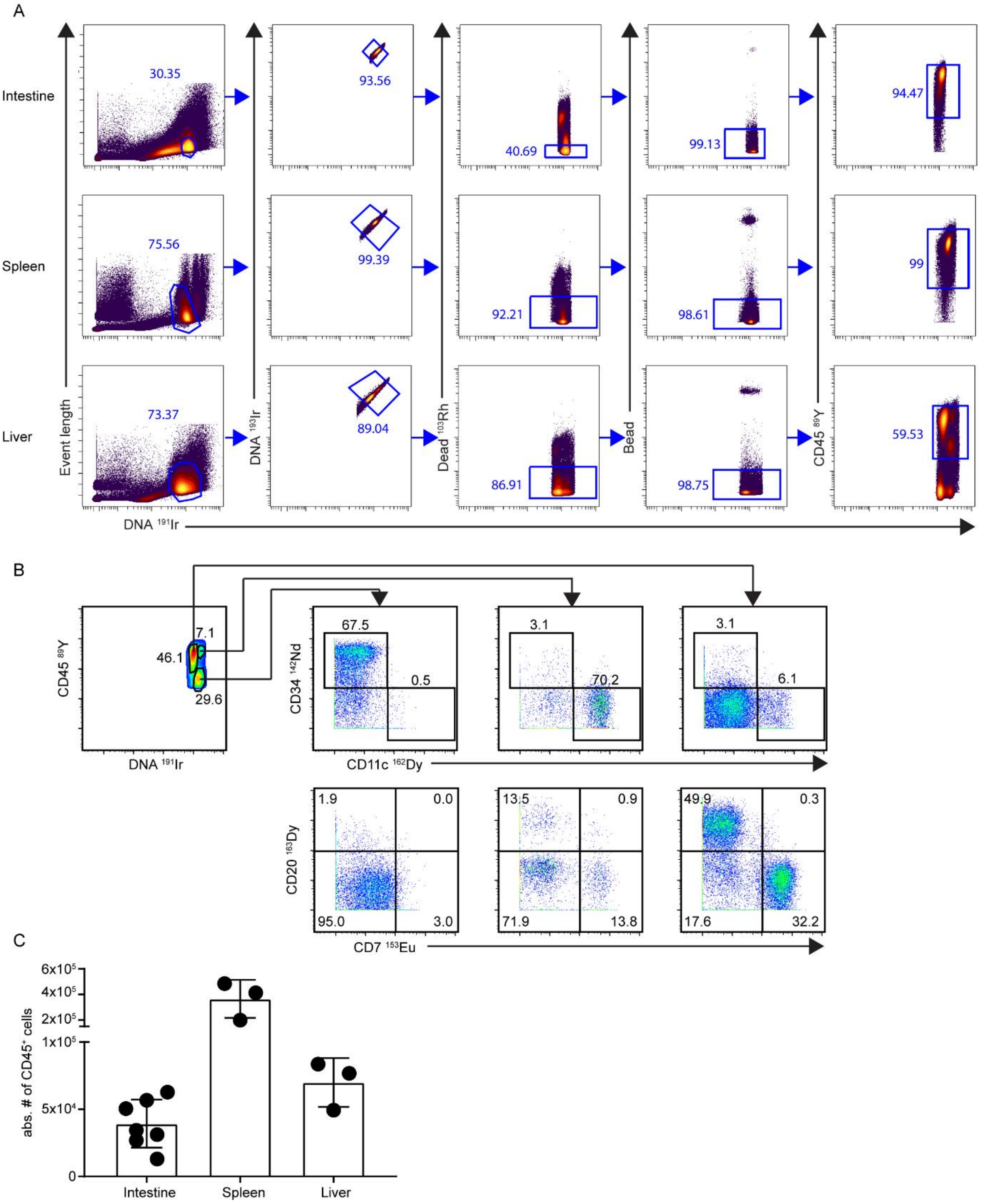

Figure S1 Gating strategy for single, live CD45^{+} cells across fetal tissues

(A) Representative biaxial plots from a fetal intestine (top panel, N =7), a fetal spleen (middle panel, N =3) and a fetal liver (bottom panel, N=3) depicting the sequential gates for single, live CD45^{+} cells with percentages. (B) Representative biaxial plots from a fetal liver (N = 3) showing the expression of CD34, CD11c, CD3 and CD20 on CD45^{low}DNA^{hi}, CD45^{hi}DNA^{hi} and CD45^{hi}DNA^{low} populations. Numbers indicated the percentages of gated cells. (C) Absolute number of single, live CD45^{+} cells acquired for each sample.

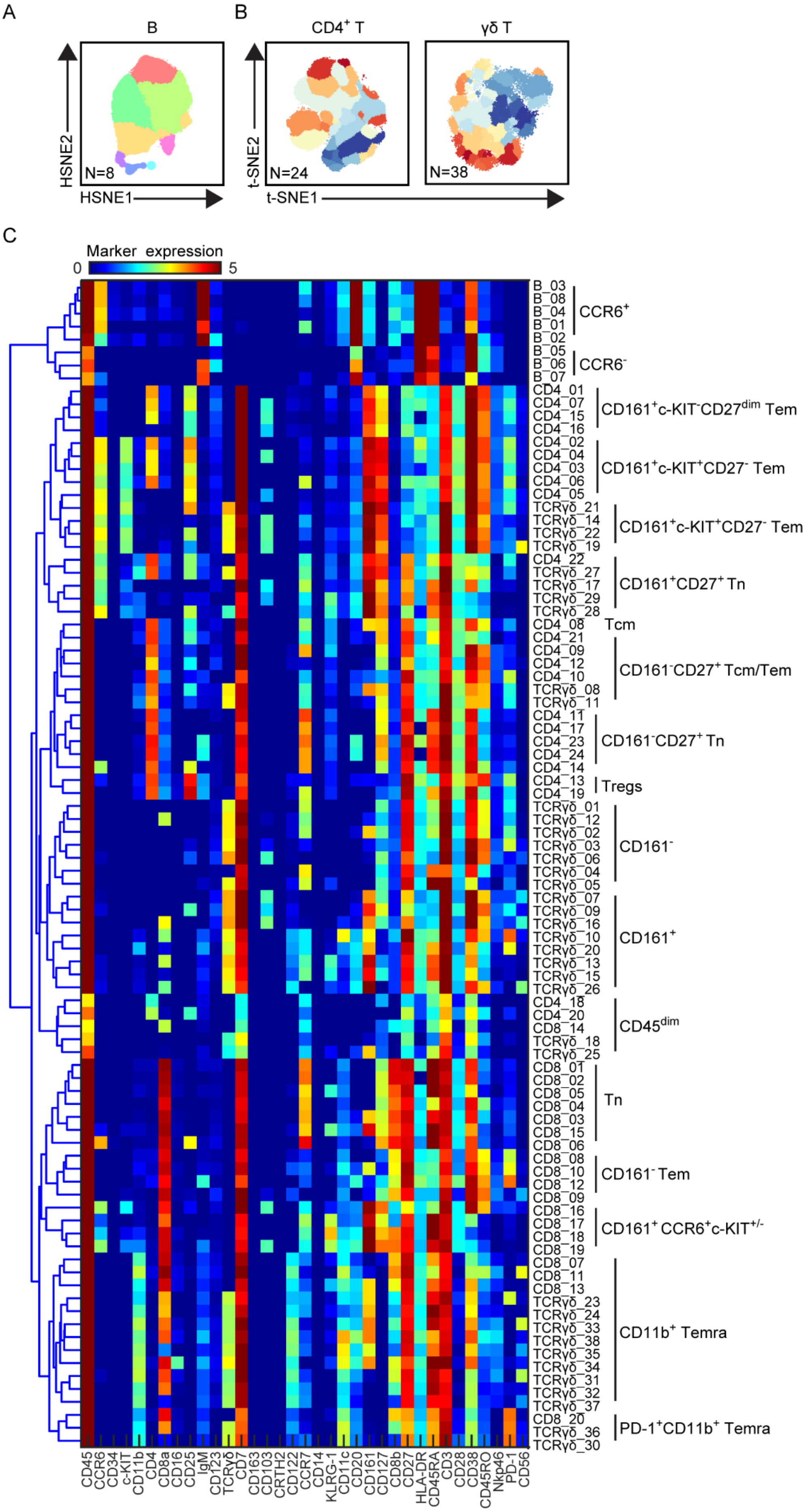
A
B
HSNE2
HSNE1
N=8
B
CD4$^+$ T
γδ T
t-SNE2
t-SNE1
N=24
N=38
C
Marker expression
0
5
B_03
B_08
B_04
B_01
B_02
B_05
B_06
B_07
CD4_01
CD4_07
CD4_15
CD4_16
CD4_02
CD4_04
CD4_03
CD4_06
CD4_05
TCRγδ_21
TCRγδ_14
TCRγδ_22
TCRγδ_19
CD4_22
TCRγδ_27
TCRγδ_17
TCRγδ_29
TCRγδ_28
CD4_08
CD4_21
CD4_09
CD4_12
CD4_10
TCRγδ_08
TCRγδ_11
CD4_11
CD4_17
CD4_23
CD4_24
CD4_14
CD4_13
CD4_19
TCRγδ_01
TCRγδ_12
TCRγδ_02
TCRγδ_03
TCRγδ_06
TCRγδ_04
TCRγδ_05
TCRγδ_07
TCRγδ_09
TCRγδ_16
TCRγδ_10
TCRγδ_20
TCRγδ_13
TCRγδ_15
TCRγδ_26
CD4_18
CD4_20
CD8_14
TCRγδ_18
TCRγδ_25
CD8_01
CD8_02
CD8_05
CD8_04
CD8_03
CD8_15
CD8_06
CD8_08
CD8_10
CD8_12
CD8_09
CD8_16
CD8_17
CD8_18
CD8_19
CD8_07
CD8_11
CD8_13
TCRγδ_23
TCRγδ_24
TCRγδ_33
TCRγδ_38
TCRγδ_35
TCRγδ_34
TCRγδ_31
TCRγδ_32
TCRγδ_37
CD8_20
TCRγδ_36
TCRγδ_30
CCR6$^+$
CCR6$^-$
CD161$^+$c-KIT$^-$CD27^{dim} Tem
CD161$^+$c-KIT$^+$CD27$^-$ Tem
CD161$^+$c-KIT$^+$CD27$^-$ Tem
CD161$^+$CD27$^+$ Tn
Tcm
CD161$^-$CD27$^+$ Tcm/Tem
CD161$^-$CD27$^+$ Tn
Tregs
CD161$^-$
CD161$^+$
CD45^{dim}
Tn
CD161$^-$ Tem
CD161$^+$ CCR6$^+$c-KIT$^{+/-}$
CD11b$^+$ Temra
PD-1$^+$CD11b$^+$ Temra
CD45
CCR6
CD34
c-KIT
CD11b
CD4
CD8a
CD16
CD25
IgM
CD123
TCRγδ
CD7
CD163
CD103
CRTH2
CD122
CCR7
CD14
KLRG-1
CD11c
CD20
CD161
CD127
CD8b
CD27
HLA-DR
CD45RA
CD3
CD28
CD38
CD45RO
Nkp46
PD-1
CD56

9

Figure S2 Analysis of the adaptive immune system across human fetal tissues using mass cytometry

(A) HSNE embedding of B cells (8.2 x 10^5 cells) at the second level. Colors indicate cluster partitions. (B) t-SNE embedding of $CD8^+$ T (6.9 x 10^4 cells) and γδ T cells (3.6 x 10^4 cells) at the single-cell level. Colors indicate cluster partitions. (C) Heatmap showing the median marker expression values of the 90 clusters identified in B, $CD4^+$ T, $CD8^+$ T and γδ T cells from seven fetal intestines, three spleens and three livers, and hierarchical clustering thereof. Tem = effector memory T cells, Tcm = central memory T cells and Temra = terminally differentiated T cells.

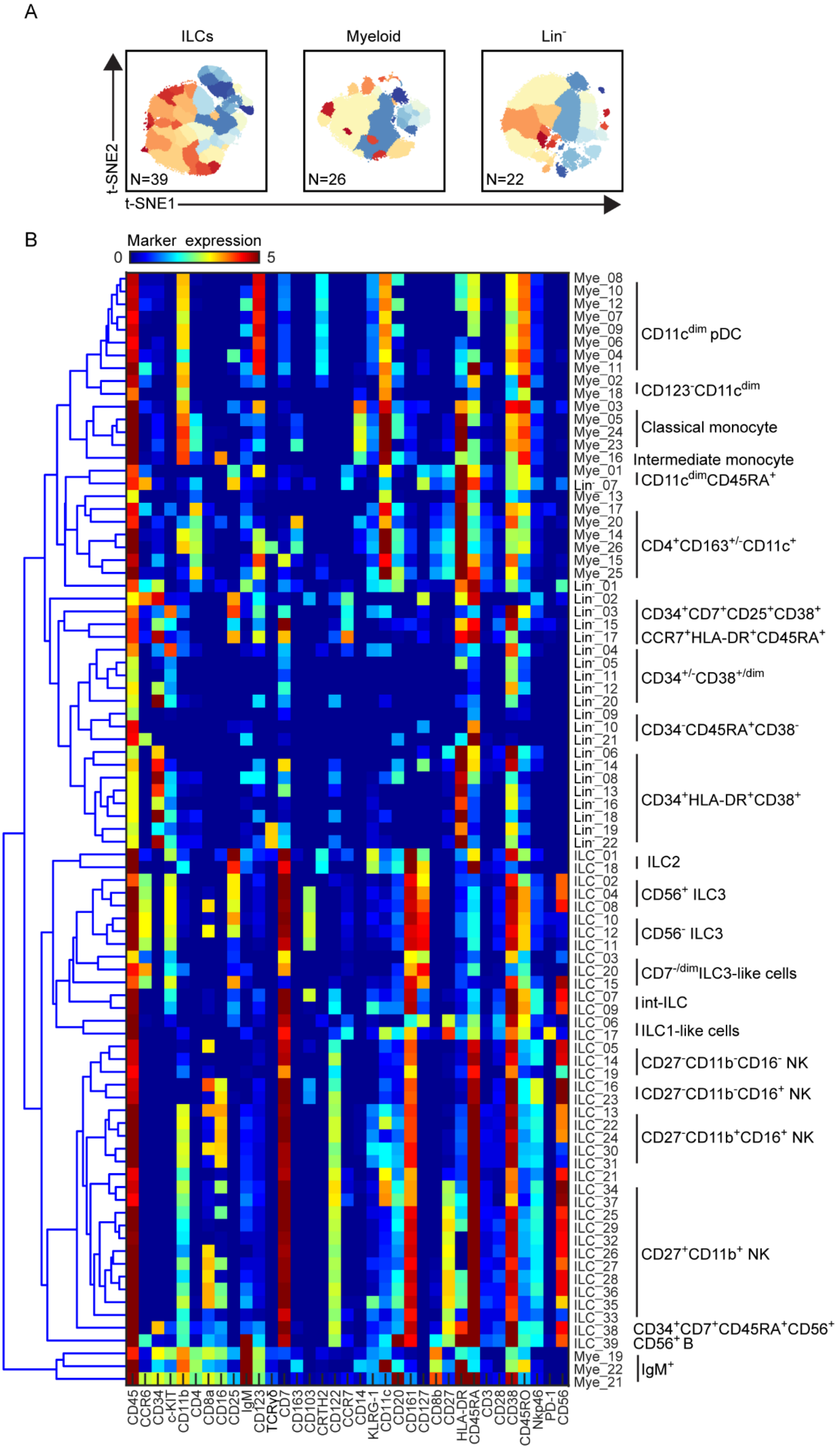

A
ILCs
Myeloid
Lin^{-}
t-SNE2
t-SNE1
N=39
N=26
N=22
B
Marker expression
0
5
Mye_08
Mye_10
Mye_12
Mye_07
Mye_09
Mye_06
Mye_04
Mye_11
Mye_02
Mye_18
Mye_03
Mye_05
Mye_24
Mye_23
Mye_16
Mye_01
Lin⁻_07
Mye_13
Mye_17
Mye_20
Mye_14
Mye_26
Mye_15
Mye_25
Lin⁻_01
Lin⁻_02
Lin⁻_03
Lin⁻_15
Lin⁻_17
Lin⁻_04
Lin⁻_05
Lin⁻_11
Lin⁻_12
Lin⁻_20
Lin⁻_09
Lin⁻_10
Lin⁻_21
Lin⁻_06
Lin⁻_14
Lin⁻_08
Lin⁻_13
Lin⁻_16
Lin⁻_18
Lin⁻_19
Lin⁻_22
ILC_01
ILC_18
ILC_02
ILC_04
ILC_08
ILC_10
ILC_12
ILC_11
ILC_03
ILC_20
ILC_15
ILC_07
ILC_09
ILC_06
ILC_17
ILC_05
ILC_14
ILC_19
ILC_16
ILC_23
ILC_13
ILC_22
ILC_24
ILC_30
ILC_31
ILC_21
ILC_34
ILC_37
ILC_25
ILC_29
ILC_32
ILC_26
ILC_27
ILC_28
ILC_36
ILC_35
ILC_33
ILC_38
ILC_39
Mye_19
Mye_22
Mye_21
$CD11c^{dim}$ pDC
$CD123^{-}CD11c^{dim}$
Classical monocyte
Intermediate monocyte
$CD11c^{dim}CD45RA^{+}$
$CD4^{+}CD163^{+/-}CD11c^{+}$
$CD34^{+}CD7^{+}CD25^{+}CD38^{+}$
$CCR7^{+}HLA\text{-}DR^{+}CD45RA^{+}$
$CD34^{+/-}CD38^{+/dim}$
$CD34^{-}CD45RA^{+}CD38^{-}$
$CD34^{+}HLA\text{-}DR^{+}CD38^{+}$
ILC2
$CD56^{+}$ ILC3
$CD56^{-}$ ILC3
$CD7^{-/dim}$ILC3-like cells
int-ILC
ILC1-like cells
$CD27^{-}CD11b^{-}CD16^{-}$ NK
$CD27^{-}CD11b^{-}CD16^{+}$ NK
$CD27^{-}CD11b^{+}CD16^{+}$ NK
$CD27^{+}CD11b^{+}$ NK
$CD34^{+}CD7^{+}CD45RA^{+}CD56^{+}$
$CD56^{+}$ B
IgM^{+}
CD45
CCR6
CD34
c-KIT
CD11b
CD4
CD8a
CD16
CD25
IgM
CD123
TCRγδ
CD7
CD163
CD103
CRTH2
CD122
CCR7
CD14
KLRG-1
CD11c
CD20
CD161
CD127
CD8b
CD27
HLA-DR
CD45RA
CD3
CD28
CD38
CD45RO
Nkp46
PD-1
CD56

Figure S3 Analysis of the innate immune system across human fetal tissues using mass cytometry

(A) t-SNE embedding of ILCs (2.6 x 10^5 cells), myeloid cells (6.4 x 10^4 cells) and Lin^- (8.7 x 10^4 cells) at the single-cell level. Colors indicate cluster partitions. (B) Heatmap showing the median marker expression values of the 87 clusters identified in ILCs, myeloid cells and Lin^- from seven fetal intestines, three spleens and three livers, and hierarchical clustering thereof. pDC = plasmacytoid dendritic cells.

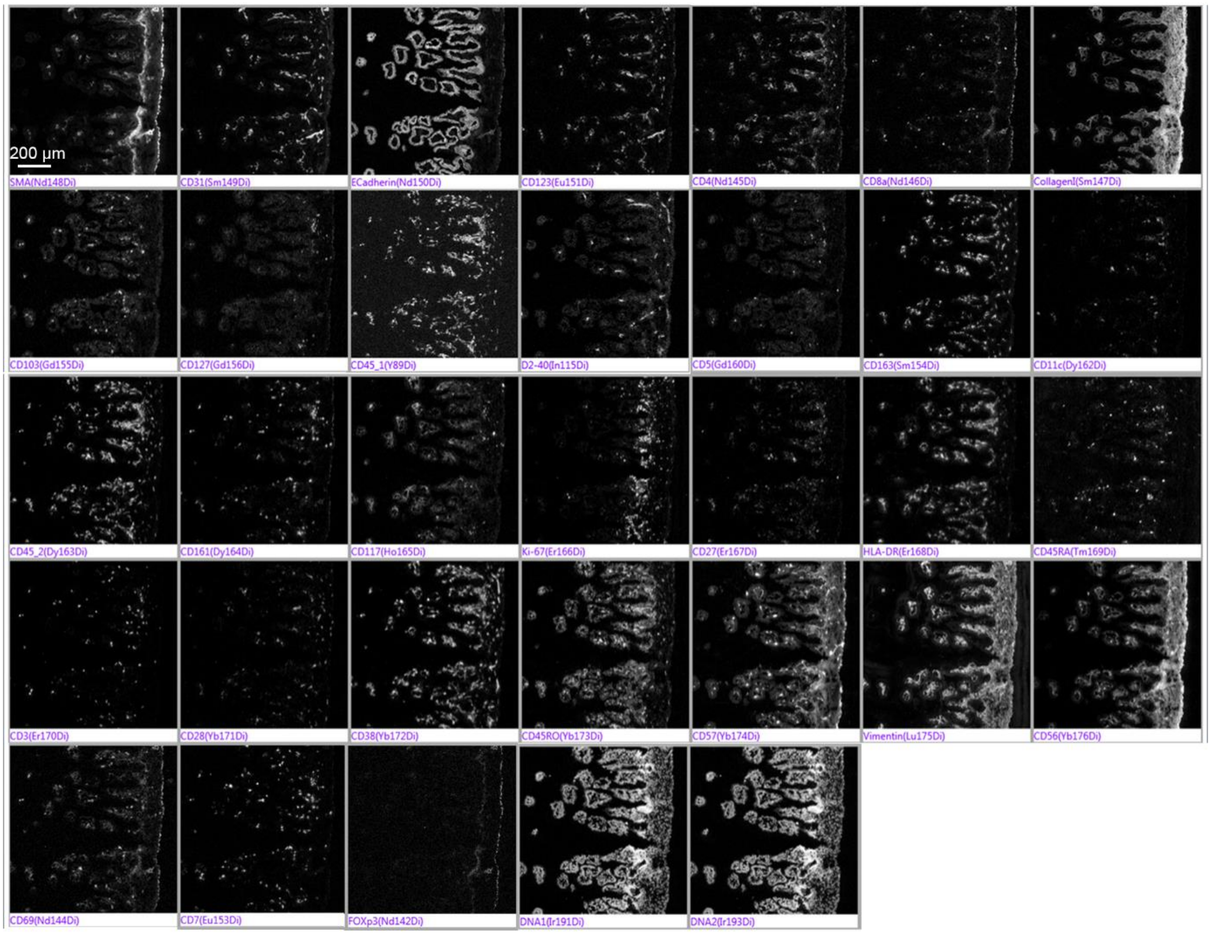

Figure S4 Validation of the imaging mass cytometry antibody panel on human fetal intestine. Representative mass cytometry images of a human fetal intestine showing the expression of the indicated markers. Scale bar: 200 µm. Scale bars are identical in all the images.

Table S1 Suspension mass cytometry antibody panel

	Antigen	Tag	Clone	Supplier	Cat.	Final dilution
1	CD127	^{165}Ho	AO19D5	Flui	3165008B	1/800
2	CCR6	^{141}Pr	G034E3	Flui	3141003A	1/200
3	CD8a	^{146}Nd	RPA-T8	Flui	3146001B	1/200
4	CD11c	^{162}Dy	Bu15	Flui	3162005B	1/200
5	CD38	^{172}Yb	HIT2	Flui	3172007B	1/200
6	CD45	^{89}Y	HI30	Flui	3089003B	1/100
7	CD117	^{143}Nd	104D2	Flui	3143001B	1/100
8	CD4	^{145}Nd	RPA-T4	Flui	3145001B	1/100
9	CD16	^{148}Nd	3G8	Flui	3148004B	1/100
10	CD25	^{149}Sm	2A3	Flui	3149010B	1/100
11	CD123	^{151}Eu	6H6	Flui	3151001B	1/100
12	CD7	^{153}Eu	CD7-6B7	Flui	3153014B	1/100
13	CD163	^{154}Sm	GHI/61	Flui	3154007B	1/100
14	CCR7	^{159}Tb	G043H7	Flui	3159003A	1/100
15	CD14	^{160}Gd	M5E2	Flui	3160001B	1/100
16	CD161	^{164}Dy	HP-3G10	Flui	3164009B	1/100
17	CD27	^{167}Er	O323	Flui	3167002B	1/100
18	CD45RA	^{169}Tm	HI100	Flui	3169008B	1/100
19	CD3	^{170}Er	UCHT1	Flui	3170001B	1/100
20	PD-1	^{175}Lu	EH 12.2H7	Flui	3175008B	1/100
21	CD56	^{176}Yb	NCAM16.2	Flui	3176008B	1/100
22	CD11b	^{144}Nd	ICRF44	Flui	3144001B	1/100
23	TCRγδ	^{152}Sm	11F2	Flui	3152008B	1/50
24	HLA-DR	^{168}Er	L243	BioL	307651	1/200
25	CD20	^{163}Dy	2H7	BioL	302343	1/200
26	CD34	^{142}Nd	HIB19	BioL	343531	1/100
27	IgM	^{150}Nd	MHM88	BioL	314527	1/100
28	CD103	^{155}Gd	Ber-ACT8	BioL	350202	1/100
29	CRTH2	^{156}Gd	BM16	BioL	350102	1/100
30	CD28	^{171}Yb	CD28.2	BioL	302902	1/100
31	CD45RO	^{173}Yb	UCHL1	BioL	304239	1/100
32	CD122	^{158}Gd	TU27	BioL	339002	1/50
33	KLRG-1	^{161}Dy	REA261	MACS	120-014-229	1/50
34	CD8b	^{166}Er	SIDI8BEE	ebio	14-5273	1/50
35	NKp46	^{174}Yb	9E2	BioL	331902	1/40

Fluidigm (Flui), eBioscience (eBio) and Biolegend (BioL).

Table S2 Sample information

Fetus ID	Gestational age, wk	Gender	Intestine	Liver	Spleen
1	19	Male	+	-	-
2	16	Female	+	-	-
3	19	Female	+	-	-
4	19	ND	+	-	-
5	21	Female	+	+	-
6	21	Female	+	-	-
7	21	Male	+	-	+
8	21	Female	-	+	+
9	16	Male	-	+	+
10	16	Female	+	+	+

ND: not determined.

Table S3 Imaging mass cytometry antibody panel

	Antigen	Tag	Clone	Supplier	Final dilution
1	CD8a	^{146}Nd	RPA-T8	Flui	1/50
2	CD11c	^{162}Dy	Bu15	Flui	1/50
3	CD117	^{165}Ho	104D2	BioL	1/50
4	CD38	^{172}Yb	HIT2	Flui	1/50
5	CD69	^{144}Nd	FN50	Flui	1/50
6	CD45	^{89}Y	HI30	Flui	1/50
7	CD4	^{145}Nd	RPA-T4	Flui	1/50
8	SMA	^{148}Nd	1A4	CST	1/200
9	CD31	^{149}Sm	89C2	CST	1/100
10	CD123	^{151}Eu	6H6	Flui	1/50
11	CD7	^{153}Eu	CD7-6B7	Flui	1/100
12	CD163	^{154}Sm	GHI/61	Flui	1/100
13	CD161	^{164}Dy	HP-3G10	Flui	1/50
14	CD27	^{167}Er	O323	Flui	1/50
15	CD45RA	^{169}Tm	HI100	Flui	1/100
16	CD3	^{170}Er	UCHT1	Flui	1/100
17	Vimentin	^{175}Lu	D21H3	CST	1/200
18	CD56	^{176}Yb	NCAM16.2	Flui	1/100
19	FOXp3	^{142}Nd	D6O8R	CST	1/100
20	CD5	^{160}Gd	UCHT2	BioL	1/25
21	HLA_DR	^{168}Er	L243	BIoL	1/600
22	E-cadherin	^{150}Nd	24E10	CST	1/50
23	CD103	^{155}Gd	EPR4166(2)	Abcam	1/50
24	CD127	^{156}Gd	R34-34	Beckman	1/50
25	CD45	^{163}Dy	D9M81	CST	1/100
26	CD28	^{171}Yb	CD28.2	BioL	1/50
27	CD45RO	^{173}Yb	UCHL1	BioL	1/50
28	Ki-67	^{166}Er	D3B5	CST	1/200
29	CD57	^{174}Yb	HNK-1/Leu-7	Abcam	1/100
30	Collagen I	^{147}Sm	polyclonal	Millipore	1/100
31	D2-40	^{115}In	D2-40	BioL	1/50
Fluidigm (Flui) and Biolegend (BioL).					

Figure R1

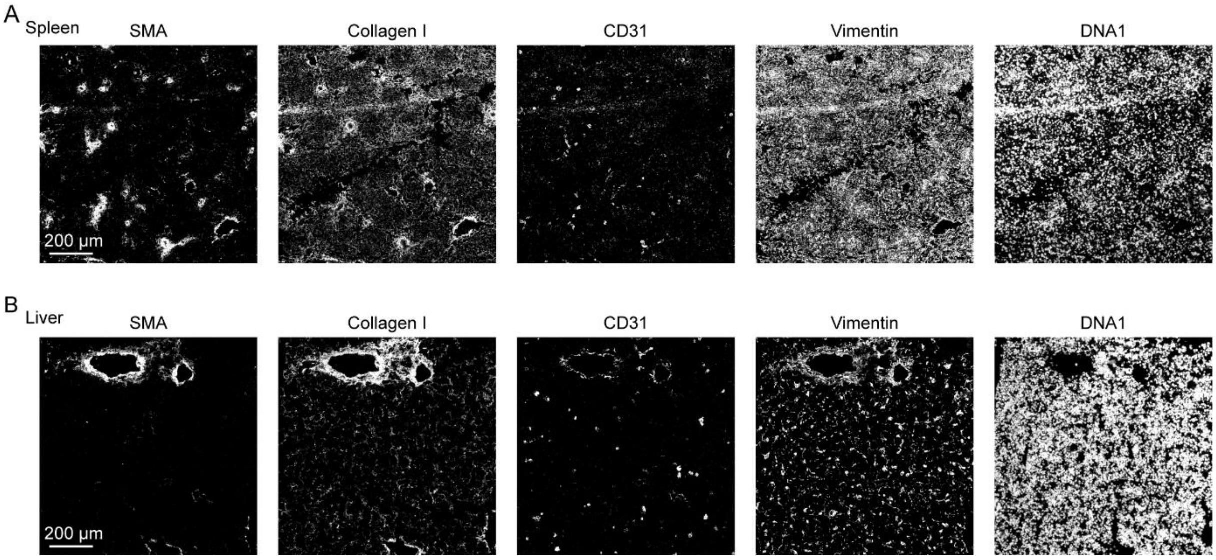

Figure R1. Expression of the indicated structural markers in the fetal spleen (A) and liver (B).

Figure R2

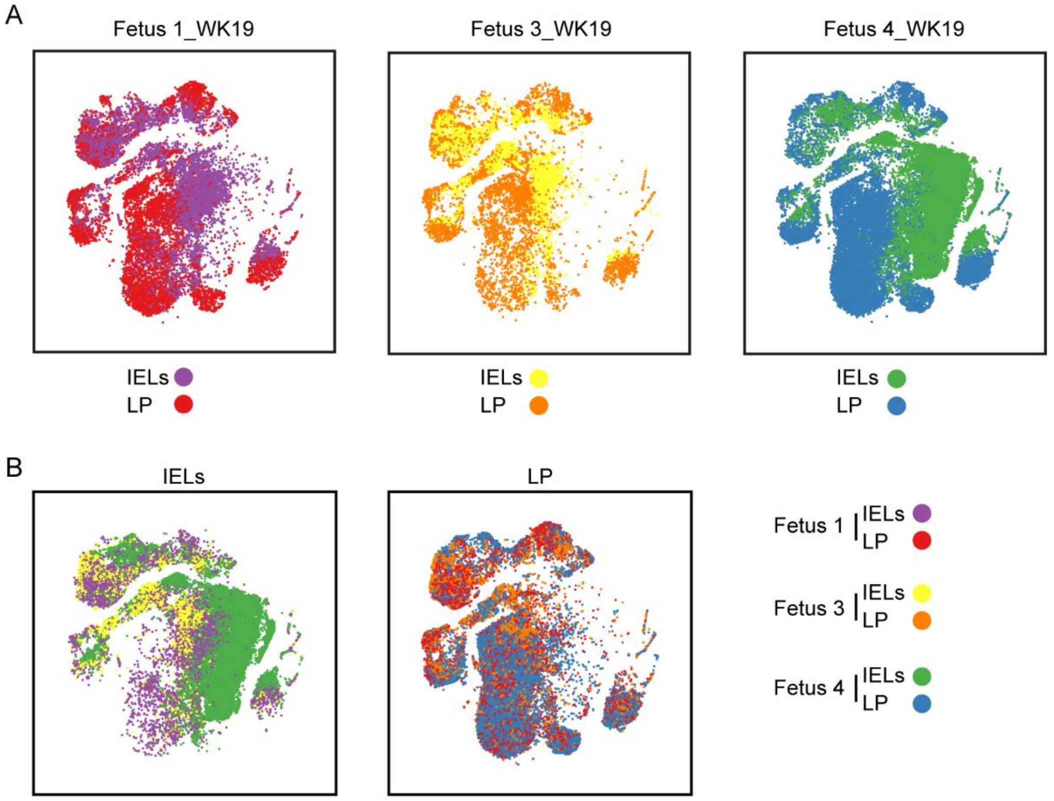

Figure R2. Collective t-SNE analysis reveals different immune signatures between epithelium and lamina propria in the human fetal intestine.

(A) t-SNE embeddings showing the $CD45^+$ immune cells derived from epithelium (IELs) and lamina propria (LP) from each human fetal intestines (N = 3). The samples of lamina propria have been downsampled to the same cell number as the paired epithelium. (B) t-SNE embeddings showing the $CD45^+$ immune cells derived from either all epithelium or all lamina propria.

Figure R3

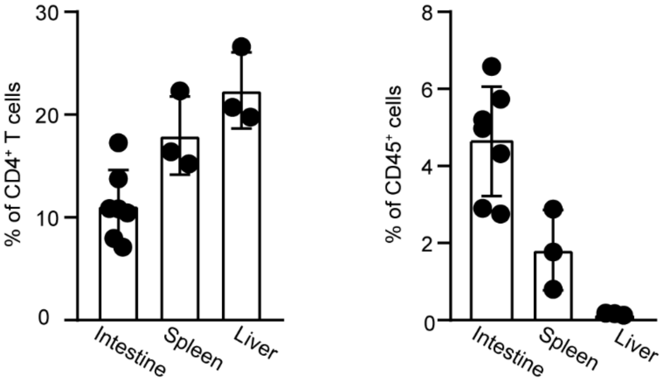

Figure R3. Distribution of Tregs across tissues.

Bar graphs showing the cell frequencies of Tregs among both $CD4^+$ T cells (left panel) and $CD45^+$ cells (right panel). Bar graphs showing mean ± SD.
